# Supplementary material for: A Novel Oral Syringe for Dosing and Administration of Multiparticulate Formulations: Acceptability Study in Preschool and School Children
Source: Pharmaceutics. 2020 Aug 25;12(9):806. doi: 10.3390/pharmaceutics12090806 (PMC7557978; doi:10.3390/pharmaceutics12090806)
Supplement: Supplementary file 1 [file pharmaceutics-12-00806-s001.pdf]

pharmaceutics

MDPI

Article

Supplementary Materials: A Novel Oral Syringe for Dosing and Administration of Multiparticulate Formulations: Acceptability Study in Preschool and School Children

Justyna Katarzyna Hofmanová, Joanne Bennett, Alastair Coupe, Jeremy A. Bartlett, Andrew Monahan and Hannah Katharine Batchelor

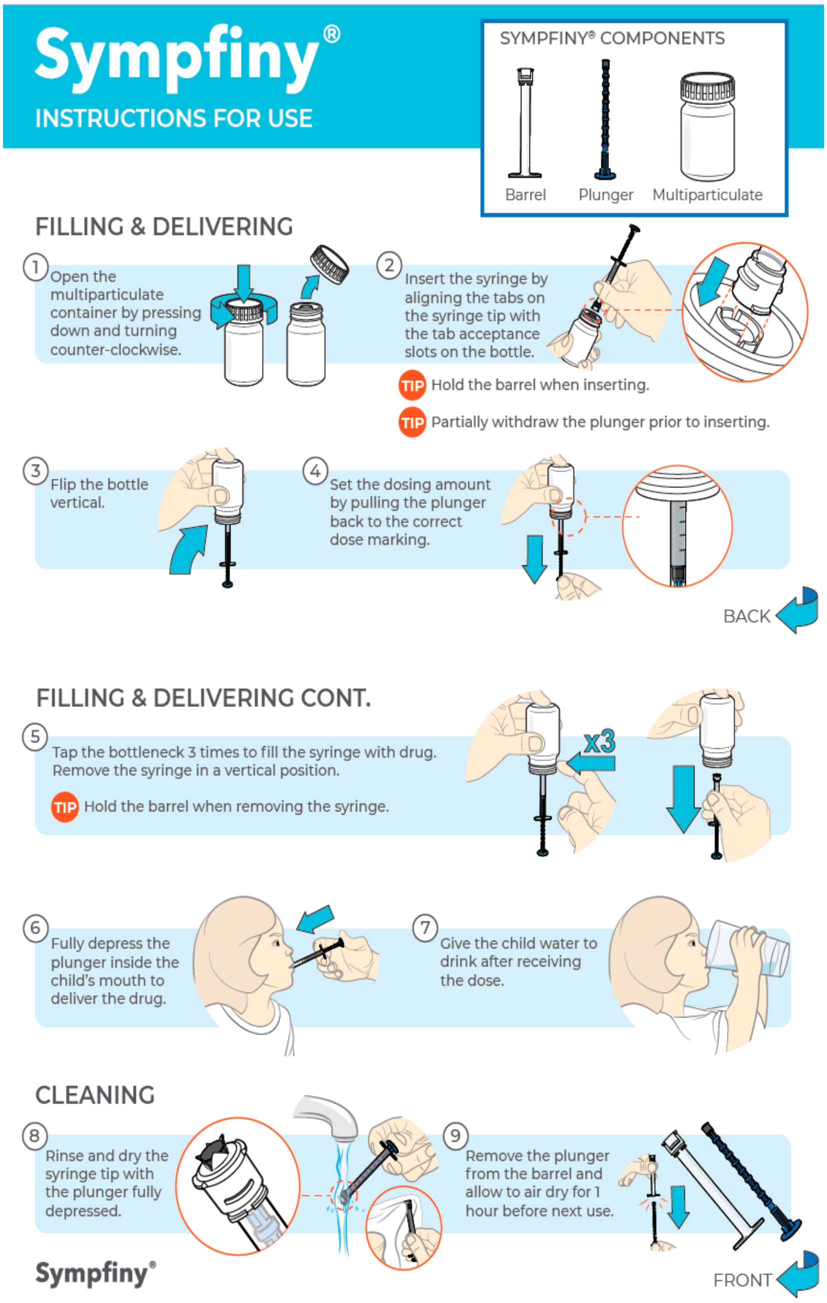

Figure S1. Sympfiny™ 'Instructions for use' presented to the study participants.
